# Alpha cell dysfunction in type 1 diabetes is independent of a senescence program

**DOI:** 10.3389/fendo.2022.932516

**Published:** 2022-10-07

**Authors:** Gabriel Brawerman, Vasilis Ntranos, Peter J. Thompson

**Affiliations:** ^1^ Department of Physiology & Pathophysiology, Rady Faculty of Health Sciences, University of Manitoba, Winnipeg, MB, Canada; ^2^ Diabetes Research Envisioned and Accomplished in Manitoba (DREAM) theme, Children’s Hospital Research Institute of Manitoba, Winnipeg, MB, Canada; ^3^ Department of Epidemiology and Biostatistics, University of California San Francisco, San Francisco, CA, United States; ^4^ Diabetes Center, University of California San Francisco, San Francisco, CA, United States

**Keywords:** alpha cells of pancreatic islet, senescence, glucagon secretion, Type 1 diabetes (T1D), DNA damage response (DDR)

## Abstract

Type 1 Diabetes (T1D) is caused by insulin deficiency, due to progressive autoimmune destruction of pancreatic β cells. Glucagon-secreting α cells become dysfunctional in T1D and contribute to pathophysiology, however, the mechanisms involved are unclear. While the majority of β cells are destroyed in T1D, some β cells escape this fate and become senescent but whether α cell dysfunction involves a senescence program has not been explored. Here we addressed the question of whether α cells become senescent during the natural history of T1D in the non-obese diabetic (NOD) mouse model and humans. NOD mice had several distinct subpopulations of α cells, but none were defined by markers of senescence at the transcriptional or protein level. Similarly, α cells of human T1D donors did not express senescence markers. Despite the lack of senescence in α cells *in vivo*, using a human islet culture model, we observed that DNA damage-induced senescence led to alterations in islet glucagon secretion, which could be rescued by inhibiting the senescence-associated secretory phenotype (SASP). Together our results suggest that α cell dysfunction in T1D is not due to activation of a senescence program, however, senescent β cell accumulation in the islet microenvironment may have a negative effect on α cell function.

## Introduction

Type 1 Diabetes (T1D) is a metabolic disease of insulin deficiency, resulting from selective autoimmune destruction of pancreatic β cells (1). While β cell loss and dysfunction are a major focus of understanding T1D pathology (2, 3), it has long been appreciated that dysfunctional glucagon secretion from α cells contributes to the disease as well (4). Indeed, reduced glucagon secretion in response to hypoglycemia and increased post-prandial glucagon secretion are frequently found in T1D patients (5, 6). Insufficient glucagon during hypoglycemia may increase risk of acute shock, while inappropriately elevated post-prandial glucagon may exacerbate hyperglycemia. Understanding the mechanisms underlying α cell dysfunction in T1D may provide important therapeutic targets for improving glycemic control and reducing risk of acute complications in people with T1D (7).

Previous work has shown that absolute insulin secretion from T1D donors is dramatically reduced as measured from both native pancreas slices (8) and isolated islets (9), consistent with dramatically reduced β cell mass. However, the overall profile of the insulin secretory response, when normalized for reduced insulin content (β cell numbers) is remarkably preserved from isolated islets of T1D donors (9, 10). Similarly, patch-clamp electrophysiology studies on isolated islet cells from T1D donors have confirmed the normal functional properties of survivor β cells (11). In contrast, isolated islets from T1D donors consistently show impaired glucagon secretion, despite no changes in α cell numbers or glucagon content relative to controls (9). A recent study also showed that dysfunctional glucagon secretion occurs in the absence of any changes in insulin secretion or β cell number in isolated islets from single autoantibody-positive donors (12), representing the earliest stages of T1D. Human α cells mis-express key transcription factors and show altered expression of genes involved in glucose metabolism, endoplasmic reticulum (ER) stress, electrical activity and calcium signaling in T1D (9), which may underlie the observed functional impairment (11). But other molecular mechanisms contributing to α cell dysfunction in T1D have not yet been investigated.

Cellular senescence is a form of permanent growth arrest, often involving an immunogenic senescence-associated secretory phenotype (SASP) and a prosurvival phenotype (13) and has been implicated in the pathogenesis of both T1D and Type 2 Diabetes (T2D) (14, 15). Our previous work found that a subpopulation of senescent β cells accumulates during the development of T1D in nonobese diabetic (NOD) mice and humans and expresses DNA damage response (DDR) marker gamma-H2A.X, cyclin-dependent kinase inhibitors Cdkn1a (p21) and Cdkn2a (p16^INK4A^), SASP markers and a Bcl-2-mediated prosurvival phenotype (14). However, whether α cells also become senescent was not explored. Senescence is a mechanism that could negatively impact α cell function either in a cell autonomous fashion if α cells activate a senescence program or in a non-cell autonomous fashion *via* SASP factor secretion from neighboring senescent β cells.

In this study, we addressed the question of whether α cells acquire features of senescence during T1D using studies in NOD mice and human donor specimens. We found that while α cells showed other markers of dysfunction and stress, they did not express a senescence program. Depletion of senescent β cells in NOD mice also did not impact the numbers of Gcg^+^ islet cells. We also addressed whether senescence affects islet glucagon secretion using our DNA damage-induced senescence human islet culture model. Surprisingly, senescence induction in isolated islets resulted in alterations in glucagon secretion, resembling what occurs in islets of autoantibody-positive and T1D donors. Despite the inter-individual variability in this phenotype, we found these changes could be rescued using small molecule inhibition of the SASP. Taken together these results suggest that while α cell senescence is not a general feature of T1D in mice or humans, the accumulation of senescent β cells in islets and secretion of SASP factors during T1D may have a negative effect on glucagon secretion by α cells.

## Materials and methods

### Human donor islets and pancreas sections

Human islets for research were provided by the Alberta Diabetes Institute and from the Integrated Islet Distribution Program (IIDP). Islets were provided from the Alberta Diabetes Institute IsletCore at the University of Alberta in Edmonton (www.bcell.org/adi-isletcore) with the assistance of the Human Organ Procurement and Exchange (HOPE) program, Trillium Gift of Life Network (TGLN), and other Canadian organ procurement organizations. Islet isolation was approved by the Human Research Ethics Board at the University of Alberta (Pro00013094). All donors’ families gave informed consent for the use of pancreatic tissue in research. For islets from the IIDP, this study used data from the Organ Procurement and Transplantation Network (OPTN). The OPTN data system includes data on all donor, wait- listed candidates, and transplant recipients in the US, submitted by the members of the OPTN. The Health Resources and Services Administration (HRSA), U.S. Department of Health and Human Services provides oversight to the activities of the OPTN contractor. The data reported here have been supplied by UNOS as the contractor for the OPTN. The interpretation and reporting of these data are the responsibility of the author(s) and in no way should be seen as an official policy of or interpretation by the OPTN or the U.S. Government. Details of specific donors are listed in **Supplementary Table 1**. DNA damage-induced senescence was induced in human islets using our previous methods (14, 16, 17). Islets were rested for 24 h after receipt in normal islet growth media (RPMI-1640 containing 10% FBS, 5.5 mM glucose and 1% antibiotic-antimycotic). Approximately 50-100 islets were then hand-picked into wells of untreated 12 well or 6 well plates and cultured in islet media containing vehicle (0.2% DMSO) or 50 μM bleomycin for 48 h. After drug incubation, media was replaced to remove the drug and islets were cultured an additional 4 days before glucagon secretion assays or other assays. Human pancreas sections were obtained from the Network for Pancreas Organ Donors with Diabetes (nPOD) repository. Details of donors used are listed in **Supplementary Table 1**.

### Immunohistochemistry

Immunohistochemical staining of formalin-fixed paraffin-embedded pancreas sections from NOD/ShiLtJ mice (Jackson Labs) and human sections were carried out as previously (14, 16). Pancreas tissue blocks from euglycemic and hyperglycemic NOD/ShiLtJ mice at different ages and 14-week old NOD mice following a 2 week treatment with 25 mg/kg ABT-199 or vehicle (60% Phosal-P50, 30% PEG400, 10% Ethanol) twice per week, as previously (14), were a kind gift from Dr. A. Bhushan (University of California San Francisco). Slides were de-paraffinized in xylene, ethanol and two washes in water and incubated for 10 minutes in 1% peroxide. Antigen retrieval was carried out with a Tinto BioSB lab-grade pressure cooker system using the high setting (121°C, 15 min) followed by permeabilization with Tris-buffered saline (25 mM Tris pH 7.4, 150 mM NaCl) containing 0.1% Triton-X-100 for 5-10 minutes. Sections were blocked with 1% normal donkey serum in protein blocking buffer (Abcam) for 15 minutes followed by primary antibody dilution in antibody diluent (DAKO) and incubation at 4°C overnight. Antibodies used for IHC are listed in **Supplementary Table 2**. Primary antibodies were detected with fluorophore conjugated secondaries and mounted with ProLong Diamond Antifade mounting media with or without DAPI (ThermoFisher). Slides were imaged on a Zeiss Axioscope 2 microscope.

### Western blot analysis

Western blot analysis was carried out as previously (17), with minor modifications. Briefly human islets were lysed in radioimmunoprecipitation assay (RIPA) buffer (ThermoFisher) containing 1X protease inhibitor cocktail and 1X phosphatase inhibitor cocktail (ThermoFisher). Protein concentrations were determined using the BCA assay (ThermoFisher). Approximately 10 μg of total protein per sample was electrophoresed on precast 4-12% gradient gels and transferred to nitrocellulose membranes using the Bolt-iBlot2 system. Membranes were blocked for 1 h in 5% skim milk in TBS containing 0.1% Tween-20 (TBS-T). Membranes were probed with primary antibodies overnight at 4C (primary antibodies are listed in **Supplementary Table 2**). After washing in TBS-T, membranes were probed with HRP-conjugated secondaries for 1 h and developed by SuperSignal west pico Plus chemi-luminescence reagent (ThermoFisher). We performed 2 or 3 different exposures to X-ray film to obtain non-saturated signals. Band intensities were quantified relative to the β-Actin loading controls from n = 3 biological replicates of islets.

### Glucagon secretion assays

Glucagon secretion was measured from human islets after resting for 24 h and induction of senescence as described above. After senescence induction, four days post-drug removal, in the first set of experiments, islets were incubated 1 h in low glucose Krebs-Ringer Buffer (KRB, 128.8 mM NaCl, 4.8 mM KCl, 1.2 mM KH2PO4, 1.2 mM MgSO4, 2.5 mM CaCl2, 5 mM NaHCO3, 10 mM HEPES) containing 0.1% BSA fraction V (Millipore-Sigma) and 2 mM glucose and incubated for 1 h at 37°C and 5% CO_2_. Islets were then transferred to fresh low glucose KRB for 1 h to collect the low-glucose supernatant, followed by 1 h in high glucose KRB (containing 20 mM glucose). In the second set of experiments, islets were incubated in KRB containing 0.1% BSA, 1X essential amino acids (ThermoFisher), 1X non-essential amino acids (ThermoFisher) and 3 mM glucose as the low glucose condition followed by 16.7 mM glucose as the high glucose condition. Islet hormone content was extracted with acidified ethanol (0.15 M HCl, 95% ethanol). Samples were centrifuged at 3000g for 5 minutes and either stored at -20°C or used for ELISA immediately. Glucagon was measured with a human glucagon ELISA kit (ThermoFisher), according to the product instructions.

### Flow cytometry

Intracellular flow cytometry was performed on dissociated human islets to stain for CDKN1A (p21) and Insulin. Briefly, after induction of senescence with bleomycin or control treatment with vehicle, islets, on day 4 post-drug removal, islets were dissociated with 0.25% Trypsin-EDTA treatment for 5 minutes at 37°C, followed by quenching in human islet media. After washing in Cell Staining Buffer (BioLegend), islet cells were incubated for 30 minutes in 1X Zombie Near-IR live-dead stain (BioLegend), washed again in Cell Staining Buffer and fixed and permeabilized using an intracellular flow cytometry kit (Novus Biologicals). Primary antibodies (shown in **Supplementary Table 2**) were incubated with permeabilized cells for 30 minutes at room temperature. After incubation with Alexa 488 or Cy5-conjugated secondaries, cells were washed in Cell Staining Buffer and analyzed by Flow cytometry on an Attune acoustic focusing cytometer equipped with 488 nm and 640 nm lasers. Cells were gated as follows: total cells (FSC-A/SSC-A), singlets (FSC-W/FSC-A), live cells (Zombie Near-IR-negative), Insulin^+^ cells (Alexa 488), and CDKN1A^+^ (Cy5). Approximately 1,500-5,000 live islet cells were scored per sample, in three experiments from three different donor islet preparations (**Supplementary Table 1**).

### Single Cell RNA-seq data analysis

**Figure 1:**
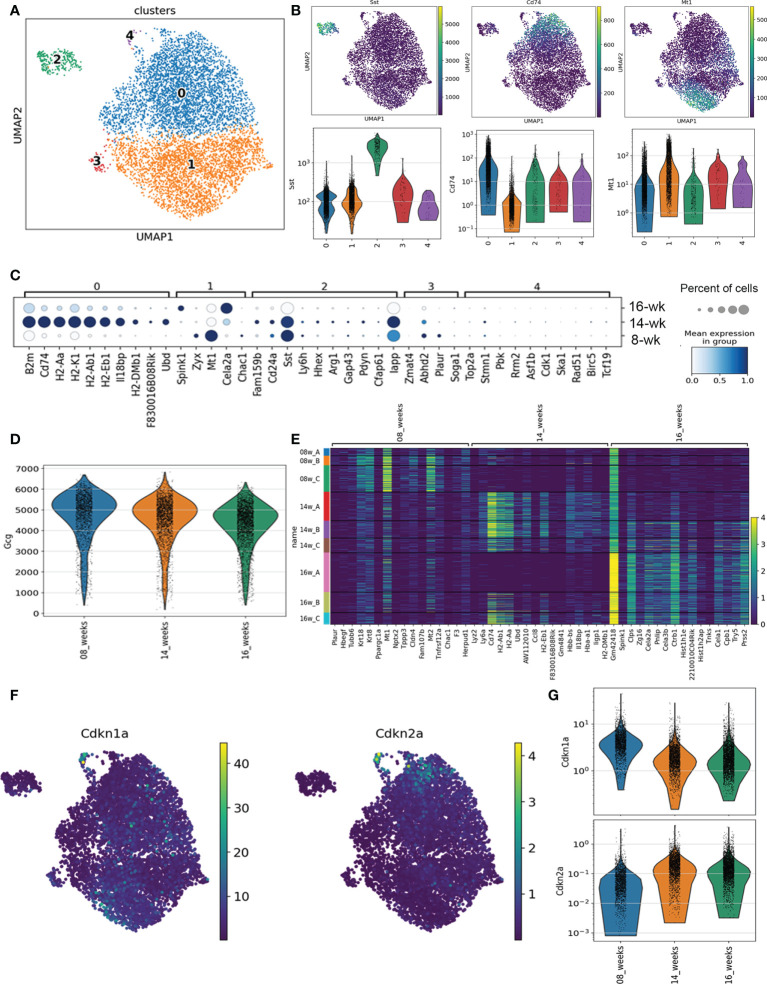
scRNA-seq analysis of α cells in NOD mice. **(A)** Dot plot transcriptional profiles of all *Gcg*-expressing cells across 8-week, 14-week and 16-week NOD mice. **(B)** Dot plot and violin plots of expression of markers of Cluster 0 (*Cd74*), Cluster 2 (*Sst*) and Cluster 1 (*Mt1*) across *Gcg*-expressing cells. **(C)** Distribution of top marker genes for each Cluster by mean gene expression level (shaded color) and percent of cells expressing the marker (circles represent bins for 20, 40, 60, 80, 100%) across all mice at each age. **(D)** Violin plot of *Gcg* expression across all identified *Gcg*-expressing cells from 8-week, 14-week and 16-week mice. **(E)** Heatmap of the top differentially expressed genes between mice at different ages. **(F)** Dot plot of senescence genes *Cdkn1a* and *Cdkn2a* across all *Gcg*-expressing cell subpopulations. **(G)** Violin plot of *Cdkn1a* and *Cdkn2a* expression across all *Gcg*-expressing cells by age.

We analyzed scRNA-seq data from CD45+ cell-depleted islet cell populations from 8-week, 14-week and 16-week euglycemic female NOD mice, which was previously published (14). The raw sequencing data were preprocessed as previously (14) according to the standard 10x Genomics workflow using Cell Ranger. We used scvi-tools (18, 19) to integrate the islet cells across all 9 samples 08w_A,B,C, 14w_A,B,C and 16w_A,B,C and selected the clusters with *Gcg* expressing cells for downstream processing (**Figure S1**). Our initial islet-level scvi model was trained on 32,563 cells using 5000 highly variable genes identified with SCANPY (seurat_v3 flavor) (20, 21). The α cell-specific scvi model was trained on the selected 7,417 cells on a set of 2500 highly variable genes specific to this population. We performed Leiden clustering on a 20 nearest neighbor graph computed from the scvi latent representation of the cells, setting the resolution parameter to 0.2 to obtain 5 clusters. For visualization we used the UMAP algorithm with the default parameters in SCANPY (min_dist=0.5, spread=1). We performed one-vs-all differential expression analysis using scvi, grouping cells either by clusters or age, and selecting genes that have estimated log-fold change > 1, Bayes factor > 2.5, and are expressed in at least 10% of cells in each cluster, or >50% cells in each age group. In all UMAP and violin plots we show scvi normalized counts with a library size parameter set to 10k. In the dot-plot (**Figure 1C**) the mean expression per group and the percentage of expressing cells are calculated using log-normalized raw gene counts and are shown in standard scale for each gene.

### Statistics and quantifications

Statistical comparisons were carried out on a minimum of n = 3 biological replicates or mice, where indicated and error bars are s.d. For human islet studies, we considered islets from one donor hand-picked into individual wells and treated in parallel as separate biological replicates and carried out different experiments on a total of 7 different donor islet preparations, including male and female (**Supplementary Table 1**). For immunohistochemistry on NOD pancreas sections, we performed quantifications from 4-6 randomly selected islets on sections on n = 3-5 mice per group from two different parts of the block. For immunohistochemistry on human pancreas sections, we imaged 3-5 randomly selected islets from n = 4 normoglycemic control and n = 4 T1D donors (**Supplementary Table 1**). Two-tailed T-tests were performed to compare two sample groups, whereas two-way ANOVAs were used on glucagon secretion assays under low and high glucose. For experiments with multiple T-tests, we carried out FDR-threshold p-value adjustments (FDR < 0.01) using the Benjamini-Hochberg method. Results were considered significant at *P* < 0.05. All statistical analyses were performed on GraphPad Prism v.9.4.1.

## Results

### Single cell RNA-seq analysis defines five different α cell subpopulations in NOD mice

To investigate the transcriptional programs of α cells during the natural history of T1D prior to the onset of overt hyperglycemia, we analyzed our previous single-cell RNA-seq (scRNA-seq) dataset on immune cell-depleted islets of 8-week, 14-week, and 16-week euglycemic NOD female mice (n = 3 mice per age group) (14). From the initial clustering analysis, each of the main endocrine islet cell types were identified by hormone gene expression and populations of α cells were clearly defined by their high expression of *Gcg* (**Figure S1**). To identify *Gcg*-expressing subpopulations independent of disease stage, we first performed clustering analysis of transcriptomes of all *Gcg*-expressing cells (7417 in total) from all mice. This revealed five subpopulations each comprising α cells from mice at all three ages (**Figure 1A**, **Figures S2A, S2B**). Clusters 0 and 1 were by far the largest populations, contributing ~95% of all α cells (**Figures 1** and **S2A**). Cluster 0 (~53% of α cells) was well defined by high expression of major histocompatibility complex (MHC) class II genes and other genes involved in immune signaling (*B2m, Cd74, H2-Aa, H2-K1, Psmb8)* (**Figures 1B, C**, **S3A**). In contrast, Cluster 1 expressed markers of metabolism and antioxidant response (*Mt1, Mt2, Zyx*) (**Figures 1B, C**, **S3A**). Clusters 2, 3 and 4 were comparatively much smaller and together were only ~5% of all α cells (**Figure S2A**). Cluster 2 (~3.6% of *Gcg*-expressing cells) showed high expression of the δ cell gene *Sst* along with other non-α cell islet genes (**Figures 1B, C**, **S3A**) suggestive of subpopulation with altered identity. Clusters 3 and 4 comprised only ~0.9% or ~0.6% of α cells, respectively (**Figure S2A**). Notably, Cluster 4 was clearly defined by markers of proliferation and cell cycle (*Top2a, Cdk1, Mki67*) suggestive of a very small proliferating α cell subpopulation, while Cluster 3 expressed genes associated with a variety of functions (*Abdh2, Plaur, Pde10a*) (**Figures 1C**, **S3A**).

To identify gene expression changes across the stages of disease progression, we next determined the differentially expressed genes in *Gcg*-expressing cells across the ages of mice. The majority of islets in 8-week NOD mice show early stages of immune infiltration (peri-insulitis) or remain intact, whereas at 14-16 weeks, most islets show destructive insulitis or are completely devoid of β cells. Interestingly, average *Gcg* expression decreased across the ages of mice (**Figure 1D**) suggestive of progressive dysfunction during disease progression. 8-week mice expressed high levels of metabolism and extracellular matrix genes, while 14 and 16-week mice expressed high levels of MHC class II and immune signaling genes, many of which were highly expressed in Cluster 0 **(** **Figures 1E**, **S3B**). As with the sub-clustering analysis, one of the 16-week mice (16A) did not share the same profile for immune signaling genes as compared with the other 16-week mice and the 14-week mice (**Figures 1E**, **S3B**) and indeed this sample transcriptome more resembled that of the 8-week samples (**Figure S2B**). This is consistent with our previous analysis of *Ins1/2*-expressing (β) cells where we found mouse 16A to exhibit a transcriptome resembling the 8-week mice (14).

Genes involved in senescence did not define any of the clusters of *Gcg*-expressing cells, nor were they among the top differentially expressed genes between the 8-, 14- and 16-week mice (**Figures S3A, S3B**). To investigate this further, we plotted expression of senescence genes *Cdkn1a* (encoding p21) and *Cdkn2a* (encoding p16^INK4A^) across all *Gcg*-expressing populations in the mice at each age to determine whether any α cell populations may be activating a senescence program. *Cdkn1a* is a key marker of a senescent β cell population apparent at 14-16 weeks in NOD mice (14). However, *Cdkn1a* was expressed highly in a only very few α cells from Cluster 4 (**Figure 1F**) and in general, its expression was lower in the *Gcg*-expressing cells from the 14- and 16-week mice as compared to the 8-week mice (**Figure 1G**). *Cdkn2a* expression was similarly highest a few of the Cluster 4 cells, but its expression increased between 8- and 14-week mice and was not changed further in 16-week mice (**Figures 1F, G**). The general increase in *Cdkn2a* but not *Cdkn1a* in α cells of 8- and 14-week mice suggestive of age-related islet cell senescence (22), which does not involve a persistent DDR, SASP or prosurvival phenotype (14). Consistent with this, there was generally very low level of SASP gene *Mmp2* expressed in α cells across the clusters and ages (**Figure S4A**). Also, we found low expression of DDR and DNA repair genes such as *Atm, Atr* and *Trp53bp1* in most α cells and the expression of these genes was not significantly different between clusters or between the different ages of mice (**Figure S4B**). Therefore, our scRNA-seq analysis suggested that while there are transcriptionally distinct subpopulations of α cells during diabetes development in NOD mice, α cells do not show signatures of a senescence program.

### α cell populations in NOD mice lack senescence markers at the protein level

**Figure 2:**
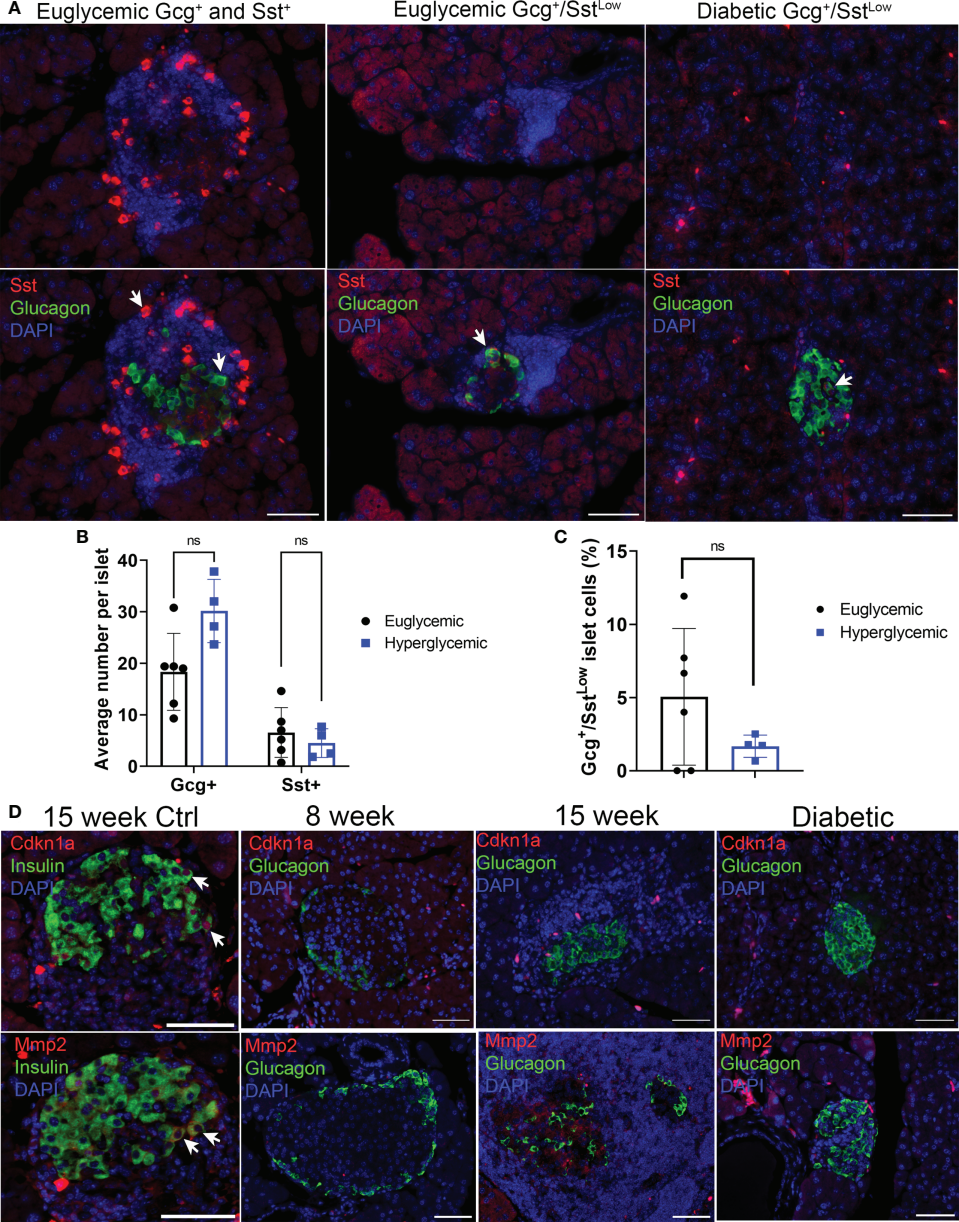
Sst but not senescence markers Cdkn1a and Mmp2 are expressed in Gcg+ cells in pancreas sections of NOD mice. **(A)** Representative immunohistochemistry staining for Sst and Gcg on pancreas sections from 14-15-week-old euglycemic and diabetic female NOD mice. White arrows indicate examples of either Gcg+ and Sst+ cells in euglycemic mice (first panel), or Gcg^+^/Sst^Low^ co-stained cells in euglycemic and diabetic mice. Scale bars = 50 μm. **(B)** Quantifications of average number of Gcg^+^ and Sst^+^ cells per islet in 14–20-week-old female euglycemic NOD mice (n=6) and age-matched diabetic mice (n=4). Error bars are s.d. ns = not significant by two-tailed T-tests, adjusted for multiple tests by Benjamini-Hochberg method. **(C)** Quantifications of the percent of Gcg^+^/Sst^Low^ cells per islet in 14–20-week-old female euglycemic NOD (n=6) and age-matched diabetic mice (n=4), error bars are s.d. ns = not significant. **(D)** Representative immunohistochemistry for Cdkn1a or Mmp2 with Ins in 15-week NOD pancreas as a control or Cdkn1a or Mmp2 with Gcg in NOD mice at indicated stages. Cdkn1a and Mmp2 were stained from serial sections of the same islet. White arrows indicate co-stained cells. Scale bars are 50 μm.

We next used immunohistochemistry to corroborate the markers that defined subpopulations of α cells from our scRNA-seq analysis at the protein level. We monitored Sst and Cd74 as markers of α cell Clusters 2 and 0, marking a potential *Gcg* ^+^/*Sst* ^+^ population and marking a population with increased MHC class II expression, respectively. Staining of pancreas sections from euglycemic and recently diabetic female NOD mice revealed Gcg^+^ only (α cells), Sst^+^ only (δ cells) and some rarer Gcg^+^ cells with low-level Sst staining (hereafter Gcg^+^/Sst^Low^) (**Figure 2A**). Quantifications revealed that the average numbers of Gcg^+^ and Sst^+^ cells per islet were not different between euglycemic and diabetic mice (**Figure 2B**). The percentage of Gcg^+^/Sst^Low^ cells per islet were variable between euglycemic mice, ranging from 0-12% and there were also no differences in the frequency of this population of cells in diabetic mice as compared with euglycemic mice (**Figure 2C**). In contrast, while we could clearly detect Cd74 staining in cells of the immune infiltrate as expected, Cd74 was not detected in Gcg^+^ islet cells (**Figure S5**). We next analyzed markers of senescence and SASP, Cdkn1a and Mmp2, respectively, which we previously found to be expressed in the senescent population of β cells of NOD mice (14). Cdkn1a and Mmp2 were expressed in some Ins^+^ islet cells in 15-17 week NOD mice (**Figure 2D**) as expected. However, we did not observe any Cdkn1a or Mmp2 expression in Gcg^+^ islet cells in non-infiltrated/intact islets of 8-week mice, insulitic islets in 13-19-week old euglycemic NOD mice, or in residual islets of recently diabetic (<1 week after hyperglycemia) 19-20 week NOD mice (n = 3 mice per group) (**Figure 2D**). Taken together these data confirm the that a subset of α cells express low levels of Sst and that α cells generally lack expression of senescence markers in NOD mice.

### Senolytic treatment of NOD mice does not affect distributions of Gcg^+^ and Sst^+^ islet cells

**Figure 3:**
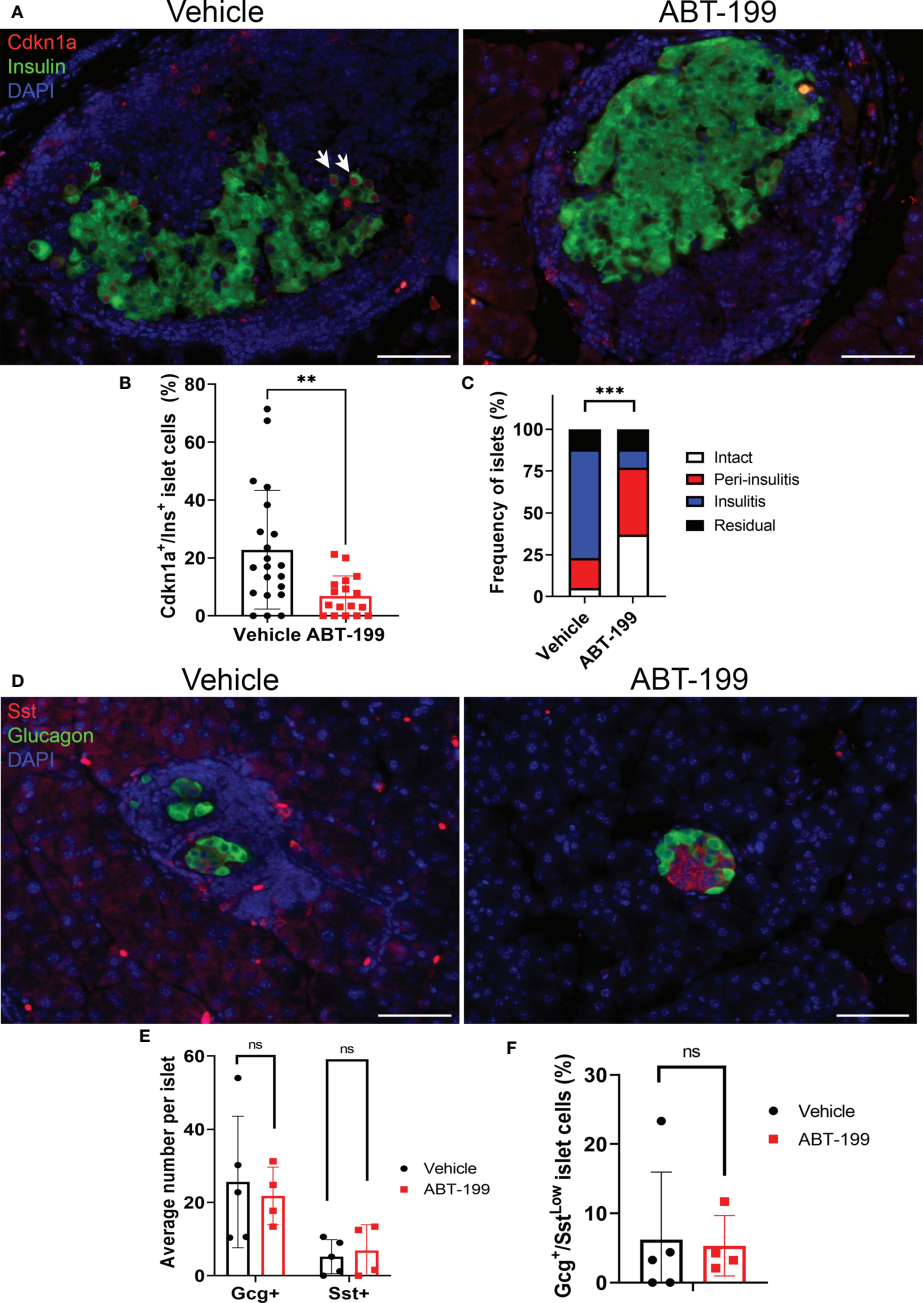
Senolytic treatment does not alter distributions of Gcg^+^, Sst^+^ or Gcg^+^/Sst^Low^ cells in NOD mice. **(A)** Representative immunohistochemistry of Cdkn1a and Ins on pancreas sections from 14-week old female NOD mice after 2 week treatment with vehicle or ABT-199. White arrows indicate examples of co-stained cells. **(B)** Quantification of the percent of Cdkn1a^+^/Ins^+^ cells per islet in islets from Vehicle-treated (n=21 islets from 4 mice) and ABT-199-treated mice (n = 17 islets from 3 mice), error bars are s.d. **P < 0.005, two-tailed T-test. **(C)** Insulitis scoring of islets from Vehicle-treated (n = 40 islets from 5 mice) and ABT-199-treated mice (n=35 islets from 4 mice). ***P < 0.0005, contingency analysis by Chi-squared test. **(D)** Representative immunohistochemistry of Gcg and Sst on pancreas sections from 14-week old female NOD mice after 2 weeks of treatment with vehicle or ABT-199. **(E)** Quantifications of average number of Gcg^+^ and Sst^+^ cells per islet in Vehicle (n=5 mice) and ABT-199-treated mice (n=4 mice) error bars are s.d. ns = not significant. **(F)** Quantifications of the percent of Gcg^+^/Sst^Low^ cells per islet in Vehicle (n=5 mice) and ABT-199-treated mice (n=4 mice), error bars are s.d. ns = not significant.

Gcg^+^/Sst^Low^ cells could represent abnormal subpopulation that arises downstream of β cell senescence. To determine whether Gcg^+^/Sst^Low^ cells were a consequence of senescent β cell accumulation during T1D development in NOD mice, we carried out immunohistochemistry for Gcg and Sst on pancreas sections from a cohort of 14-week old NOD mice that had been treated with a senolytic agent ABT-199 (n = 4 mice) or vehicle (n = 5 mice) from 12 until 14 weeks of age (**Figure 3**). ABT-199 depletes senescent β cells in NOD mice and halts progression of T1D (14). We confirmed efficient senolysis in ABT-199-treated mice, as these mice showed significantly lower average percentage of Cdkn1a^+^ β cells per islet and less severe islet lymphocyte infiltration as compared with controls (**Figures 3A–C**) consistent with arrested disease progression. Staining for Gcg and Sst revealed no significant differences in the average numbers of Gcg^+^ or Sst^+^ cells per islet or the percentage of Gcg^+^/Sst^Low^ cells per islet in ABT-199-treated mice as compared with controls (**Figures 3D–F**). These results suggest the numbers of Gcg^+^ and Sst^+^ cells are not altered by senolytic treatment and that Gcg^+^/Sst^Low^ cells are not a consequence of senescent β cell accumulation during T1D progression *in vivo*.

### Human α cells do not show evidence of senescence in T1D

**Figure 4:**
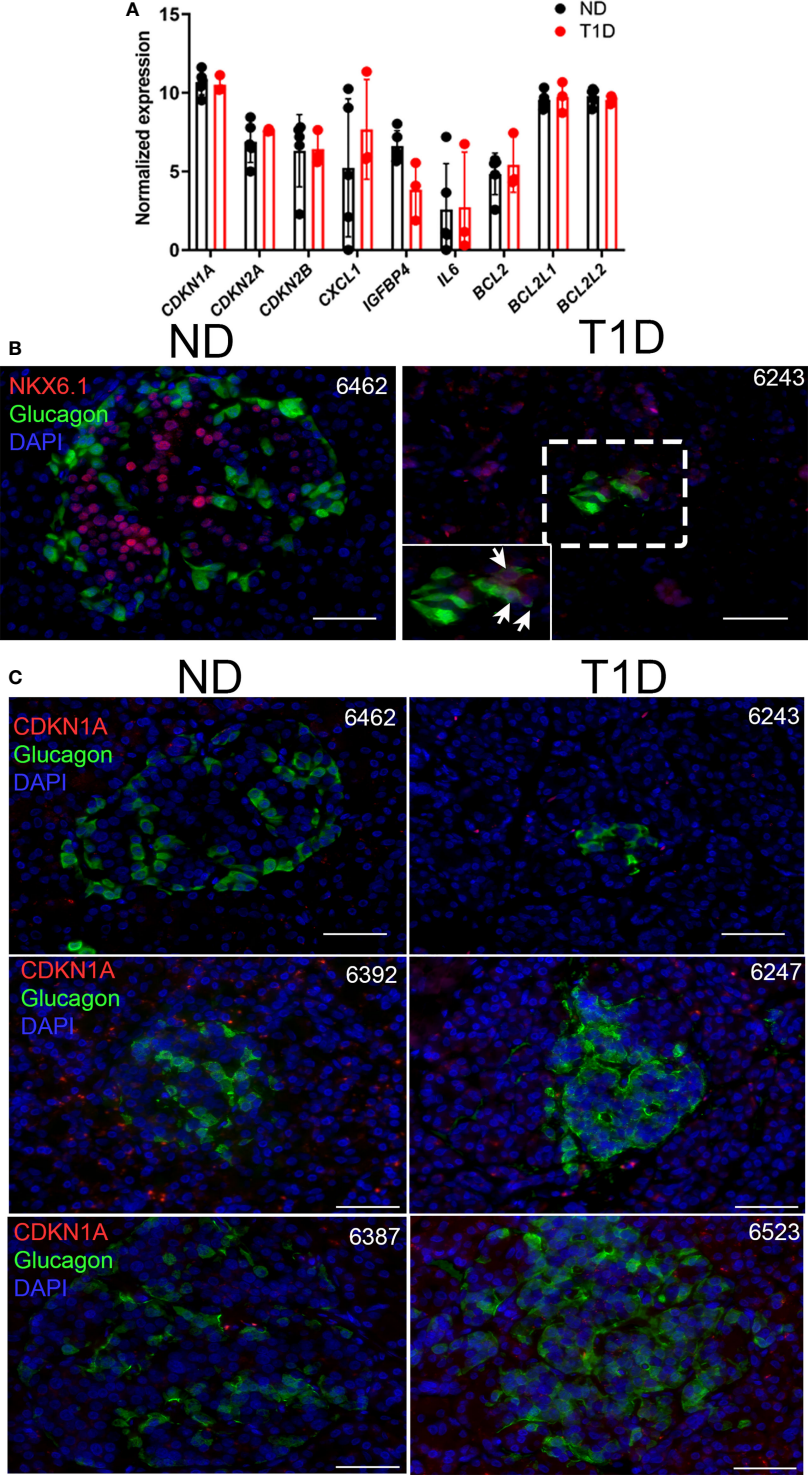
Human T1D α cells show no changes in senescence genes and do not express senescence marker CDKN1A. **(A)** Normalized RNA-seq gene expression values for islet/β cell senescence genes from publicly available dataset GSE106148 for five control no diabetes (ND) and three T1D donors. None of the differences were significant. **(B)** Representative immunohistochemistry for NKX6.1 with GCG on ND and T1D donor pancreas sections. Zoomed inset shows NKX6.1 nuclear staining in several GCG+ cells. Scale bars = 50 μm. nPOD donor IDs are listed at top right of each image. **(C)** Representative immunohistochemistry for CDKN1A with GCG from ND and T1D donor pancreas sections. Scale bars = 50 µm. nPOD donor IDs are listed at top right of each image.

Next, we explored whether human α cells acquire features of senescence in T1D. First we analyzed expression of our previously defined islet/β cell senescence and SASP genes (14, 16) in publicly available RNA-seq data from Brissova et al. (9) on sort-purified human α cells from five no diabetes control donors (ND) and three T1D donors, including one recent onset and two with established T1D (**Figure 4A**; **Supplementary Table 1**). One of the donors used in the α cell RNA-seq by Brissova et al. (donor 6342) we previously showed had accumulated senescent β cells (14). Notably, there were no differences in expression of any genes previously implicated in β cell senescence (14, 16), such as cyclin-dependent kinase inhibitor genes *CDKN1A, CDKN2A*, SASP genes *IL6*, *CXCL1, IGFBP4* or BCL-2 family prosurvival genes *BCL2, BCL2L1*, and *BCL2L2* in α cells of T1D donors relative to ND donors (**Figure 4A**). In addition, among the previously identified differentially expressed genes from that study, there were none involved in senescence and DNA damage responses (9). To determine whether α cells expressed senescence markers at the protein level, we carried out immunohistochemistry for CDKN1A with GCG on age-matched ND donor and new, recent onset and established (0.58-8 years duration) T1D donor pancreas sections (n = 4 donors per group, **Supplementary Table 1**). We selected T1D donors (ages 12-24) representing a range of residual C-peptide, from 0.04-0.47 ng/ml, reflecting a spectrum of remaining functional β cell mass (**Supplementary Table 1**). Consistent with Brissova et al. (9), we observed NKX6.1^+^/GCG^+^ islet cells in T1D donors, indicative of α cell dysfunction (**Figure 4B**). However, while CDKN1A^+^/INS^+^ islet cells were detected in T1D donors, as previously reported (14) (**Figure S6**), we did not detect any CDKN1A^+^/GCG^+^ islet cells (**Figure 4C**) (**Supplementary Table 1**). Taken together these data suggest that α cells do not acquire signatures of senescence at the transcriptional or protein levels during new, recent onset, and established T1D in humans.

### Senescence alters human islet glucagon secretion in a culture model *via* the SASP

**Figure 5:**
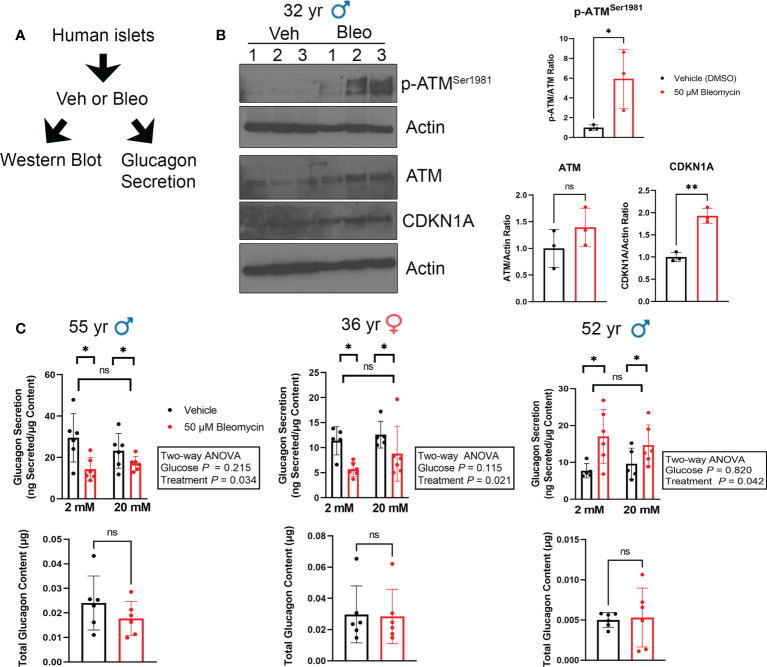
Glucagon secretion is altered during senescence induction in human islets ex vivo. **(A)** Isolated islets from nondiabetic control donors were treated in culture with 50 μM bleomycin to induce DNA damage and senescence, or with vehicle (0.15% DMSO) as a control for 48 h followed by drug-washout and culture in drug-free media for 4 days before harvesting for western blot analysis or glucagon secretion assays. **(B)** Western blot analysis of phosphorylated ATM^Ser1981^, total ATM and CDKN1A four days after bleomycin treatment to induce senescence as in **(A)**. β-Actin was a loading control. Data are mean and s.d. of n = 3 biological replicates. *p < 0.05, **p < 0.005, ns = not significant, two-tailed T-tests. **(C)** Glucagon ELISAs were carried out after sequential incubation of control and senescent islets from three different donors in low glucose (2 mM) or high glucose (20 mM). Data are means of n = 6 biological replicates (different wells of islets from the same donor), error bars are s.d and were analyzed by Two-way ANOVA as indicated.

Since we did not find expression of senescence markers in α cells during T1D in NOD mice or humans, we next explored how senescent β cell accumulation in islets affects glucagon secretion. To this end, we employed our recently characterized DNA damage-induced islet senescence model, using sub-lethal treatment of islets from ND donors with the DNA damaging agent bleomycin (17) (**Figure 5A**). This treatment leads to induction of a senescence program that recapitulates features of senescent β cells that accumulate in autoantibody-positive and T1D donors, including activated *CDKN1A* expression and SASP (14, 16). After bleomycin treatment for 48 h and culture in drug-free media for an additional 4 days, we confirmed that islets underwent senescence as evidenced by a persistent DDR marked by activated ATM and increased CDKN1A expression at the protein level (**Figure 5B**). At this time-point we monitored glucagon secretion under low (2 mM) and high (20 mM) glucose conditions from three different islet donor preparations (**Figure 5C**). We previously examined insulin secretion during senescence induction in these same islet donor preparations and found that bleomycin-induced senescence did not impact islet insulin secretion (17). Using these same samples to measure glucagon secretion, we found that senescent islets had reduced glucagon secretion under low and high glucose in two donors, whereas the third donor showed increased glucagon secretion under both conditions (**Figure 5C**). Senescence did not significantly impact total glucagon content in the islets under these conditions (**Figure 5C**) demonstrating that these changes are not likely driven by differences in α cell abundance following DNA damage.

**Figure 6:**
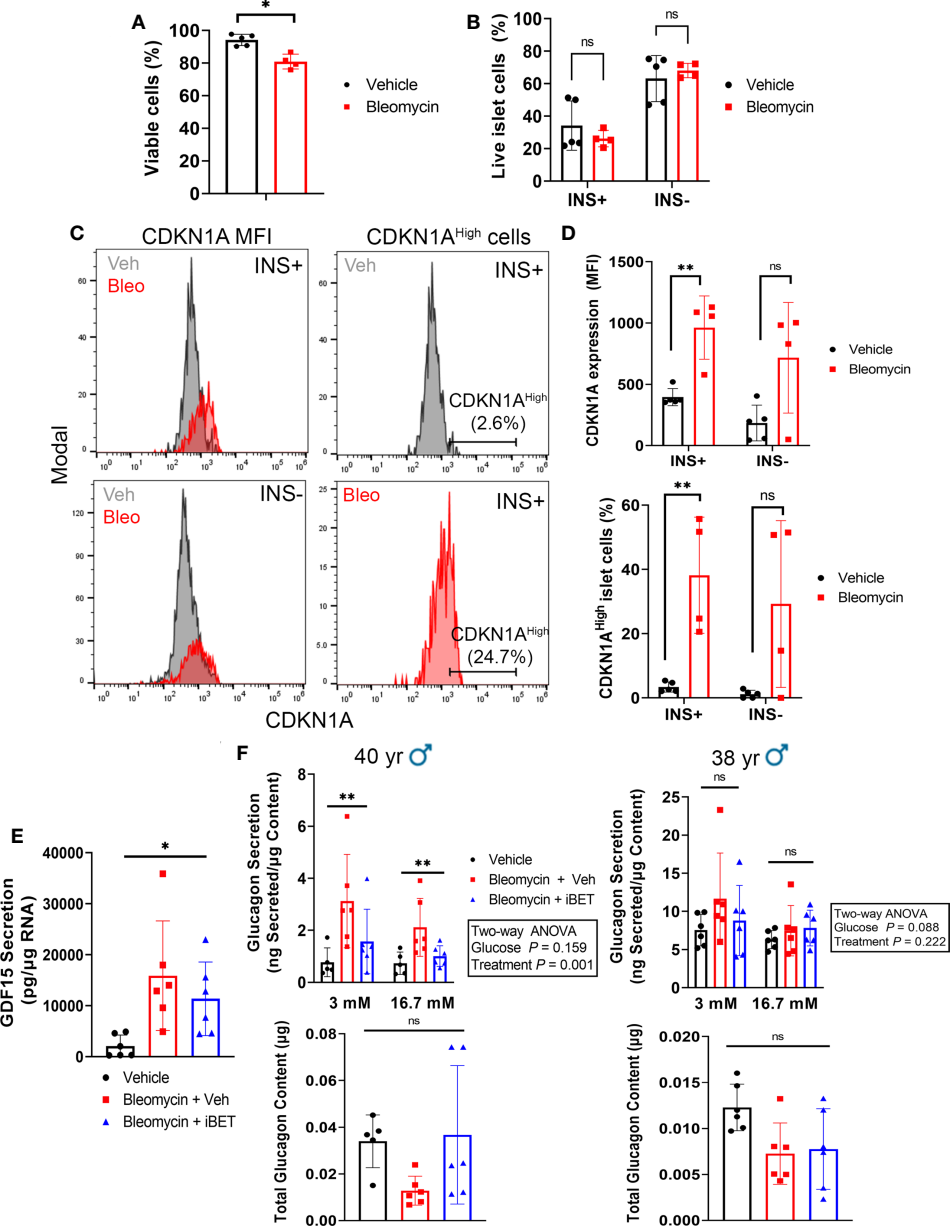
β cells consistently upregulate CDKN1A in the human islet senescence model and altered glucagon secretion during senescence can be rescued by inhibition of SASP with iBET-762. **(A)** Viability of all islet cells as measured by negativity for Zombie Near-IR dye following induction of senescence with bleomycin (50 µM) or treatment with vehicle (DMSO) control at day 4 post-drug removal. Data are mean ± s.d. from n = 5 vehicle control biological replicates or n = 4 bleomycin-induced senescence biological replicates from 3 independent human islet donors. *p < 0.05, two-tailed T-test. **(B)** Viability of INS+ and INS- islet cells following senescence induction with bleomycin or control vehicle treatment at day 4 post-drug removal, from the same samples as in **(A)**. ns = not significant, two-tailed T-tests corrected for multiple testing by Benjamini-Hochberg method. **(C)** Representative histograms depicting CDKN1A fluorescence intensity in populations of INS+ and INS- cells or the gating for the CDKN1A^High^ population of INS+ cells, each from Vehicle and Bleomycin-induced senescent islets. **(D)** Quantifications of CDKN1A median fluorescence intensity (MFI) in INS+ versus INS- cells, using same samples as in **(A)**. **p < 0.005, ns = not significant, two-tailed T-tests corrected for multiple testing by Benjamini-Hochberg method. **(E)** Luminex assay quantification of the secretion of SASP factor GDF15 from conditioned media of vehicle control or bleomycin-induced senescent islets treated with vehicle or with 5 µM iBET-762. Conditioned media was sampled at day 5 post-drug removal after 5 day incubation with vehicle or iBET-762. Data are mean ± s.d. from n = 6 vehicle control biological replicates or n = 6 bleomycin-induced senescence biological replicates from 2 independent human islet donors. *p < 0.05, one-way ANOVA. **(F)** Glucagon secretion assays and total glucagon content measured from vehicle control, bleomycin-induced senescent treated with vehicle or bleomycin-induced senescent islets treated with 5µM iBET-762, from two independent islet donors, as in **(E)**. Data are mean ± s.d. from n = 5 or 6 biological replicates from each sample group, per donor. Results of two-way ANOVAs (Glucose and Treatment) are indicated next to each graph. Glucagon content was compared between groups using one-way ANOVA, ns = not significant.

Since both α and β cells could develop senescence in our human islet model, the glucagon secretion phenotypes may be due to direct DNA damage and senescence induction in α cells. To determine whether β cells and/or non-β cells were becoming senescent in our model, we used intracellular flow cytometry to score the frequency of INS^+^ cells (β cells) versus INS^-^ (non-β cells) that expressed CDKN1A in vehicle control and bleomycin-treated islets at day 4 post-drug removal (**Figure 6**). Using islet samples from three donors we found that bleomycin treatment only led to a modest decrease in cell viability in comparison to controls (94% versus 80%, **Figure 6A**). However, on average 25-50% of live islet cells were INS^+^ whereas 50-75% were INS^-^ and senescence induction did not significantly alter the frequency of viable INS^+^ versus INS^-^ islet cells (**Figure 6B**). Bleomycin treatment led to a consistent and significant increase in CDKN1A expression in β cells, as there was a 2-3-fold increase in CDKN1A median fluorescence intensity (MFI) in the INS^+^ population (**Figure 6C**). In contrast, INS^-^ islet cells showed variable changes in CDKN1A expression after bleomycin treatment, which on the whole was not statistically significant (**Figure 6D**). The same effect was found when gating the INS^+^ or INS^-^ cells expressing the highest levels CDKN1A (CDKN1A^High^, ~2-5% of the cells in each population in the control samples) and comparing this subpopulation between bleomycin and control islets (**Figure 6C**). The percent of CDKN1A^High^ cells was consistently and significantly increased in the INS^+^ subpopulation but was variably affected in INS^-^ cells (**Figure 6D**). Given that the majority of islet cells in these experiments were INS^-^/non-β cells (50-75%) and yet a more consistent and reproducible effect of increased CDKN1A expression occurred in the β cells, these data suggest that INS^+^ β cells preferentially upregulate CDKN1A in our DNA damage-induced senescence culture model as compared to INS^-^/non-β cells.

Having observed that β cells consistently upregulate CDKN1A in our islet senescence model, we addressed whether the glucagon secretion defects were due to a paracrine effect of SASP after senescence induction. Senescent β and non-β cells in our model could exert a paracrine effect on non-senescent α cells *via* SASP factor secretion, thereby altering glucagon secretion. To test this possibility, we induced senescence in islets and after bleomycin removal during the development of SASP, we cultured islets in the Bromodomain ExtraTerminal domain (BET) protein family inhibitor iBET-762 (23), which blocks transcriptional activation and secretion of SASP factors in human islets following bleomycin-induced DNA damage and senescence (17). If the glucagon secretory phenotype was due to direct DNA damage and toxicity in α cells, then it should not be amenable to rescue by iBET-762, whereas, if it is a consequence of SASP after DNA damage, then iBET-762 should rescue the phenotype. We confirmed that iBET-762 treatment had an inhibiting effect on SASP by carrying out Luminex assays on the conditioned media from senescent and control islets, which confirmed a significant reduction in the secretion of GDF15 (**Figure 6E**), a putative SASP factor that is consistently induced in our human islet senescence model (17). We next performed secretion assays using 3 mM and 16.7 mM glucose in the presence of amino acids to more closely mimic physiological glucagon stimulation. Under these conditions we found inter-donor variability in both the glucagon secretion phenotype and impact of iBET-762. Senescent islets from a 40-year-old male donor showed elevated glucagon secretion and iBET-762 alleviated this phenotype, with a significant difference among the treatments groups (**Figure 6F**). However, only a trend in this phenotype was observed in senescent islets from a second 38-year-old male donor, which was not statistically significant (**Figure 6F**). Although there were trends, there were also no significant differences in glucagon content comparing the treatment groups in each donors (**Figure 6F**). Taken together, these data suggest that induction of senescence in our human islet culture model can have a negative impact on α cell function as a result of SASP.

## Discussion

α cell dysfunction is a significant, yet poorly understood aspect of T1D pathophysiology (7). While islets isolated from T1D donors generally preserve normal insulin secretory responses, glucagon secretion is notably impaired (9, 10). Defects in α cell function in T1D are not likely to be driven by reduced β cell mass alone, since isolated islets of single autoantibody-positive donors also show abnormal glucagon secretion, with no apparent changes in β cell mass or function (12). Gene expression profiling of α cells has revealed changes in metabolism and identity genes (9, 12) and immunohistochemistry has shown a subset of α cells in T1D donors express chemokine CXCL10 (24), suggesting they may contribute to islet inflammatory responses in T1D. However, whether dysfunction in α cells involves senescence had not been explored. Here we report evidence that α cells do not express senescence markers in T1D, contrasting with what is observed in a subpopulation of β cells in NOD mice and humans during T1D (14).

We identified several transcriptionally distinct α cell subpopulations in NOD mice. The vast majority of α cells (~95%) were marked by elevated expression of metabolism and antioxidant response genes or MHC class II and immune signaling genes, but we also found rarer populations expressing markers of altered islet identity, such as *Sst*, and a very small population expressing proliferation and cell cycle genes. A proliferative α cell population was previously identified by scRNA-seq in human islets (25) and other studies have shown upregulation of MHC Class II in islets and β cells during T1D in NOD mice and humans (26–28), consistent with our findings. While we corroborated the presence of rare Gcg^+^/Sst^Low^ islet cells, we did not detect Gcg^+^/Cd74^+^ islet cells, suggesting that this population identified by scRNA-seq analysis may not express sufficiently high transcript levels to visualize Cd74 at the protein level. In contrast, both our scRNA-seq analysis and immunostaining showed there were no α cell subpopulations defined by senescence markers in NOD mice. The Gcg^+^/Sst^Low^ cell population was not increased in diabetic mice and was not affected by senolytic treatment, suggesting these cells are not the result of metabolic stress or a consequence of senescent β cell accumulation. Similarly, we observed no changes in average numbers of α or δ cells in NOD mice treated with senolytic. Nevertheless, there may be other abnormalities of α cells that are related to the presence of senescent β cells and further studies will be required to clarify whether senescent β cell depletion affects α cell function in NOD mice. In addition to our studies in NOD mice, we found no differences in expression of key senescence genes in sort-purified human α cells in T1D donors from previous RNA-seq datasets. In fact, CDKN1A, which we found marked senescent human β cells (14), was not expressed in α cells of T1D donors. Together these studies provide evidence that α cells do not undergo a senescence program during T1D.

Our human islet senescence culture model provided us with an ex vivo system to address the question of what happens to α cell function in the context of a culture model for DNA damage and senescence induction in β cells. This model recapitulates the main features of senescent β cells that accumulate in T1D, including an upregulation of CDKN1A but not CDKN2A and SASP factor expression (14, 16). Importantly, in this culture model, following induction of DNA damage, islets are cultured in drug-free media for an additional 4 or 5 days. Therefore, the outcomes are reflective of stable phenotypic changes rather than acute responses, which we confirmed here by the persistent DDR signaling and SASP four days after exposure to DNA damage. Interestingly, senescence induction with bleomycin led to changes in glucagon secretion, resembling what was previously reported in isolated islets of T1D donors and single autoantibody-positive donors (9, 12). Despite identical treatments to induce senescence, changes in glucagon secretion clearly varied between different islet donors, pointing to the role of biological sex and genetic background in modifying the effects of senescence (17). Although we observed variable expression of CDKN1A in non-β cells following senescence induction, β cells were consistently found to upregulate CDKN1A, suggesting that they are preferentially affected in this model. It should be noted that the non-β cell population includes a variety of other cell types in addition to α cells and thus we could not directly determine whether α cells were becoming senescent in these experiments. Altered glucagon secretion during senescence induction was rescued by a small molecule inhibitor of BET proteins, iBET-762, which inhibits SASP. Again, inter-donor variability was observed in the extent of the senescence-induced changes in glucagon secretion between similarly aged, sex-matched donor islet preparations, limiting the ability to draw generalized conclusions from this initial study. Further studies using larger numbers of islet donor preparations and dynamic perifusion conditions would reveal whether there are consistent effects of senescence on islet glucagon secretion across different donors and identify features of responders versus non-responders. Consistent with the possibility for SASP to alter α cell function *in vivo*, senescent β cells accumulate at higher levels in islets of autoantibody-positive donors compared to no diabetes controls (14) and autoantibody-positive donor islets exhibit abnormally increased glucagon secretion. Additional studies will be required to investigate the paracrine mechanisms by which senescent β cells influence α cell function, which are not limited to SASP.

In conclusion, we present evidence that unlike β cells, α cells do not acquire a senescence program in T1D, despite the defects in α cell function that occur during the disease. This conclusion is consistent with previous findings showing that α cells are less sensitive than β cells to T1D-related stress, such as inflammatory cytokines (29), ER stress (30) and viral infection (31). Nevertheless, DNA damage-induced senescence leads to altered islet glucagon secretion in a culture model in a SASP-dependent manner, suggesting a negative effect of senescent islet/β cells on α cell function. This work paves the way for exploring how the accumulation of senescent β cells may negatively impact α cell function in T1D.

## Data availability statement

Publicly available datasets were analyzed in this study. This data can be found here: NCBI GEO: https://www.ncbi.nlm.nih.gov/geo/query/acc.cgi?acc=GSE117770, GSE117770 and https://www.ncbi.nlm.nih.gov/geo/query/acc.cgi?acc=GSE106148, GSE106148.

## Ethics statement

Ethical review and approval was not required for the animal study because the study used fixed animal tissue specimens provided by a third party and did not involve any studies on live animals.

## Author contributions

GB carried out all experiments with guidance from PT. VN carried out bioinformatics analyses of the scRNA-seq data. PT designed the study and wrote the manuscript. All authors contributed to the article and approved the submitted version.

## Funding

This work was funded by institutional start-up funds from the Children’s Hospital Research Institute of Manitoba and the University of Manitoba to PT. Human pancreatic islets and/or other resources were provided by the NIDDK-funded Integrated Islet Distribution Program (IIDP) (RRID : SCR_014387) at City of Hope, NIH Grant # 2UC4DK098085 and the JDRF-funded IIDP Islet Award Initiative.

## Acknowledgments

This research was performed with the support of the Network for Pancreatic Organ donors with Diabetes (nPOD; RRID : SCR_014641), a collaborative type 1 diabetes research project supported by JDRF (nPOD: 5-SRA-2018-557-Q-R) and The Leona M. & Harry B. Helmsley Charitable Trust (Grant#2018PG-T1D053, G-2108-04793). The content and views expressed are the responsibility of the authors and do not necessarily reflect the official view of nPOD. Organ Procurement Organizations (OPO) partnering with nPOD to provide research resources are listed at http://www.jdrfnpod.org/for-partners/npod-partners/.

## Conflict of interest

The authors declare that the research was conducted in the absence of any commercial or financial relationships that could be construed as a potential conflict of interest.

## Publisher’s note

All claims expressed in this article are solely those of the authors and do not necessarily represent those of their affiliated organizations, or those of the publisher, the editors and the reviewers. Any product that may be evaluated in this article, or claim that may be made by its manufacturer, is not guaranteed or endorsed by the publisher.
