# Supplementary material for: Alpha cell dysfunction in type 1 diabetes is independent of a senescence program
Source: Front Endocrinol (Lausanne). 2022 Oct 7;13:932516. doi: 10.3389/fendo.2022.932516 (PMC9586489; doi:10.3389/fendo.2022.932516)
Supplement: Supplementary file 1 [file DataSheet_1.docx]

**Supplementary Material**


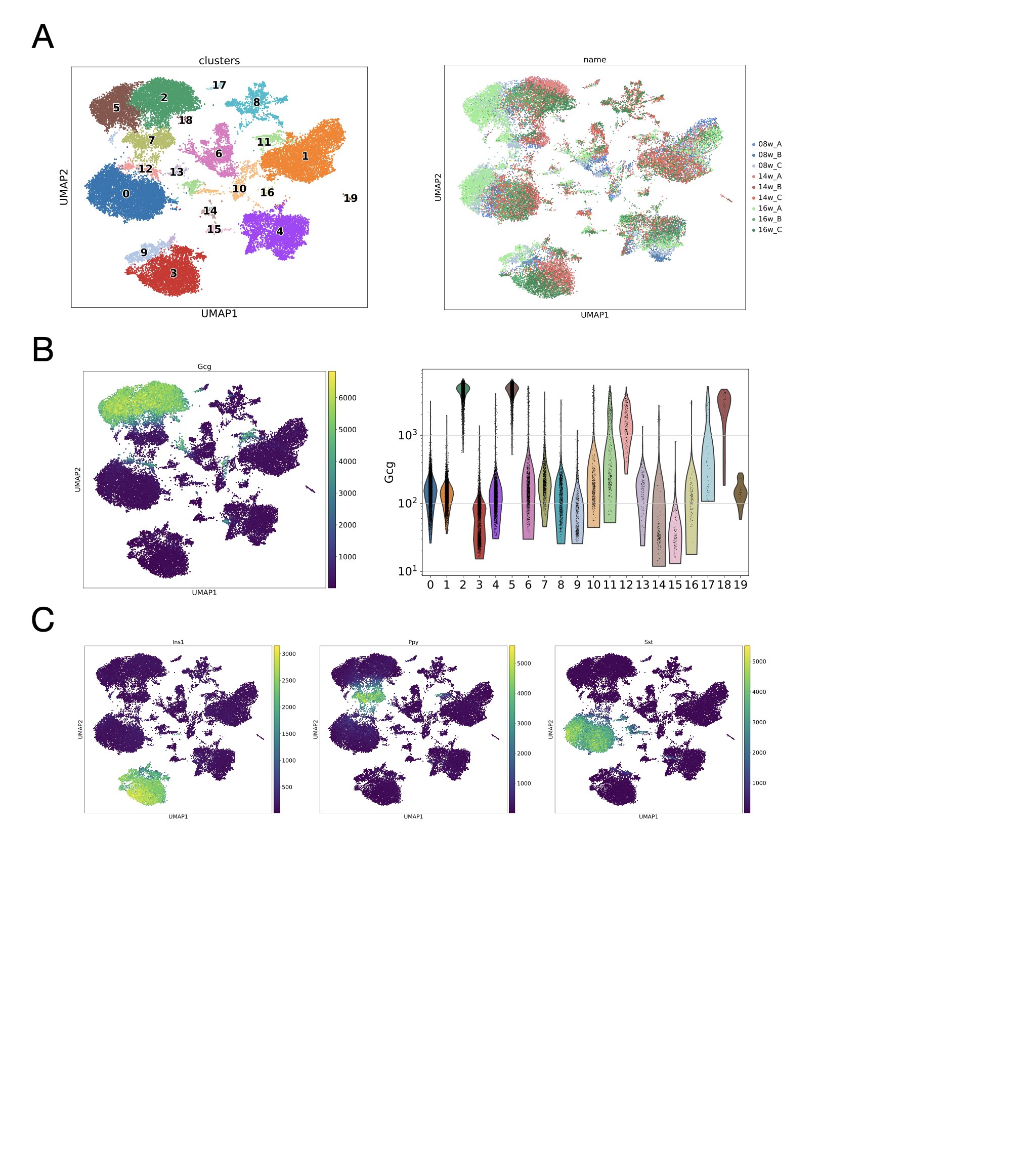


**Supplementary Figure 1. Identification of α cells and other endocrine cells from NOD mouse scRNA-seq dataset based on *Gcg* expression.** Cell populations are numbered in the first colored plot and plotted for *Gcg* expression in the violin plot. Other plots show expression of *Ins1* (β cells), *Sst* (δ cells) and *Ppy* (PP cells) in other populations and confirm the specificity of the α cell population.


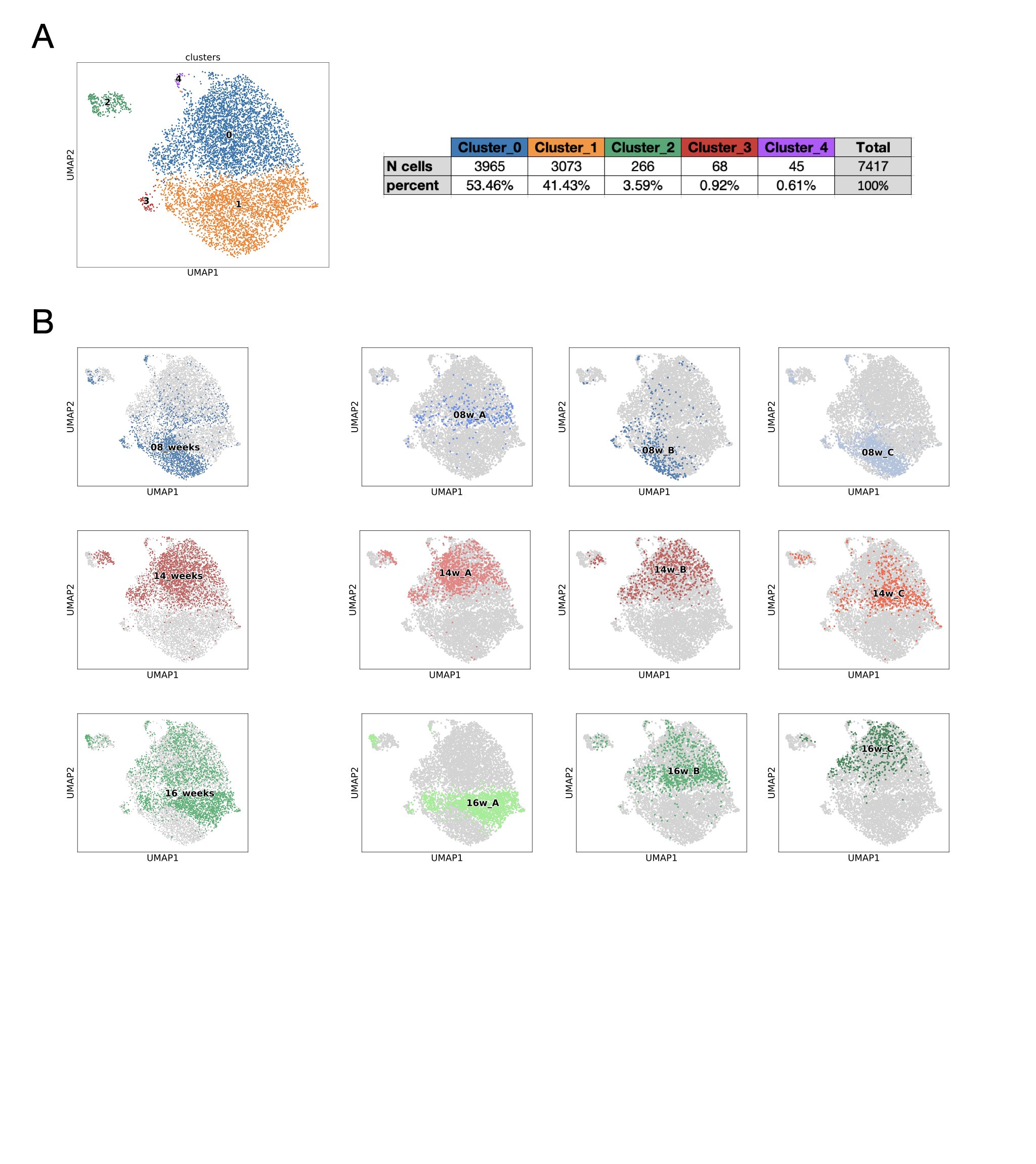


**Supplementary Figure 2. Clustering analysis of *Gcg*-expressing cells.** (A) Transcriptomes of 7417 *Gcg*-expressing cells formed 5 distinct clusters. (B) Contributions of each mouse at each age to the 5 *Gcg*-expressing subpopulations.


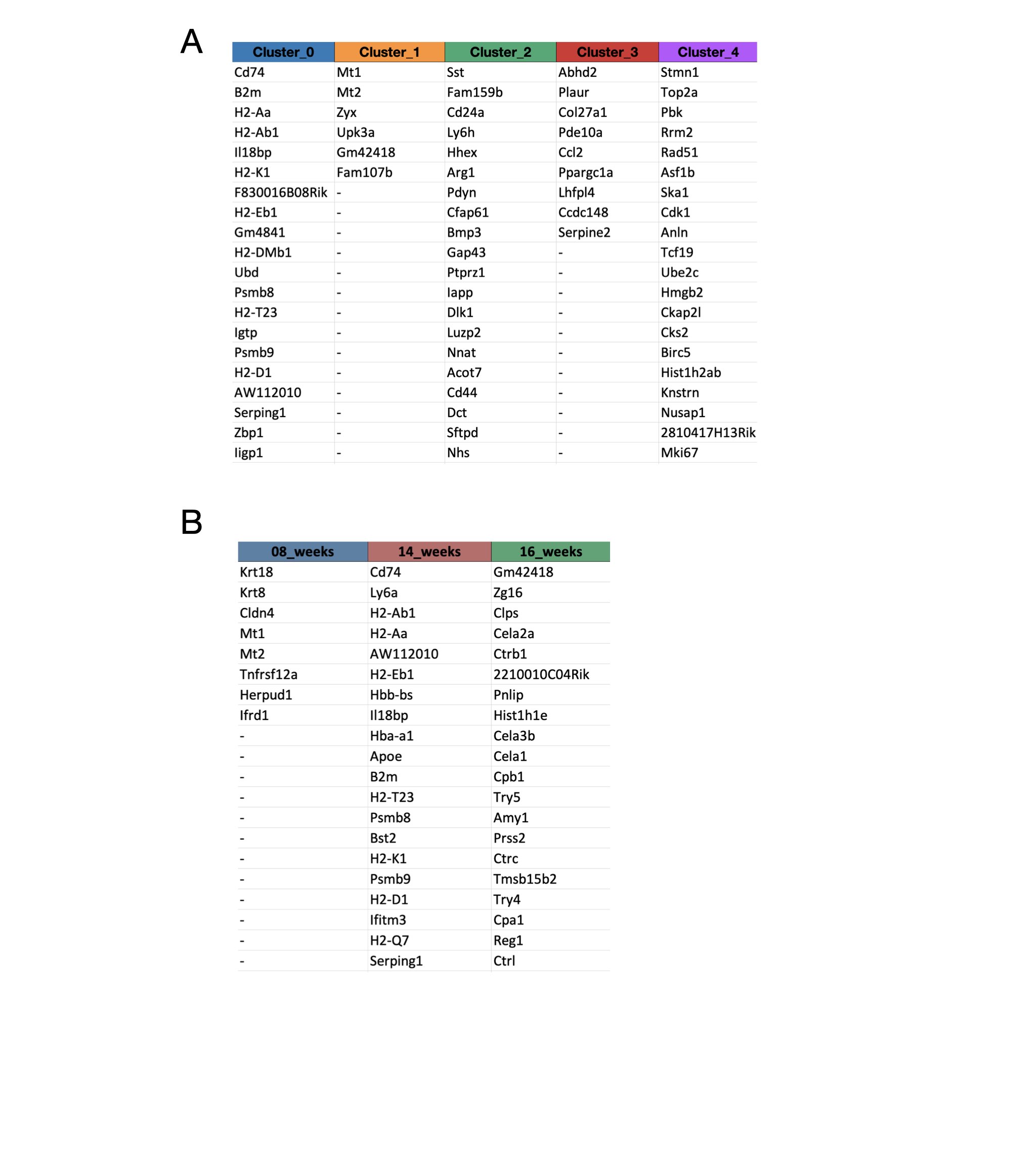


**Supplementary Figure 3. Differentially expressed genes in *Gcg*-expressing cells.** (A) Differentially expressed genes based on cluster. (B) Differentially expressed genes based on age group.

A

**
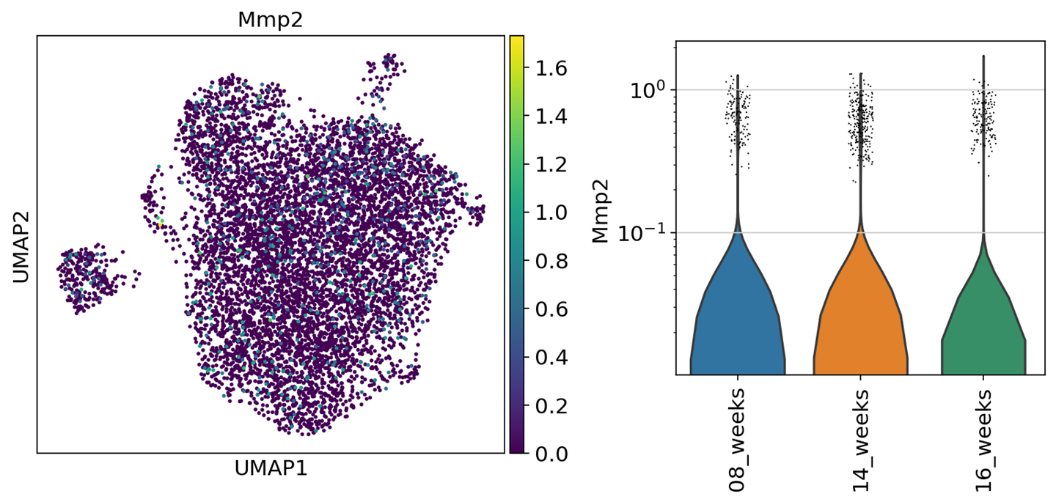
**

B

**
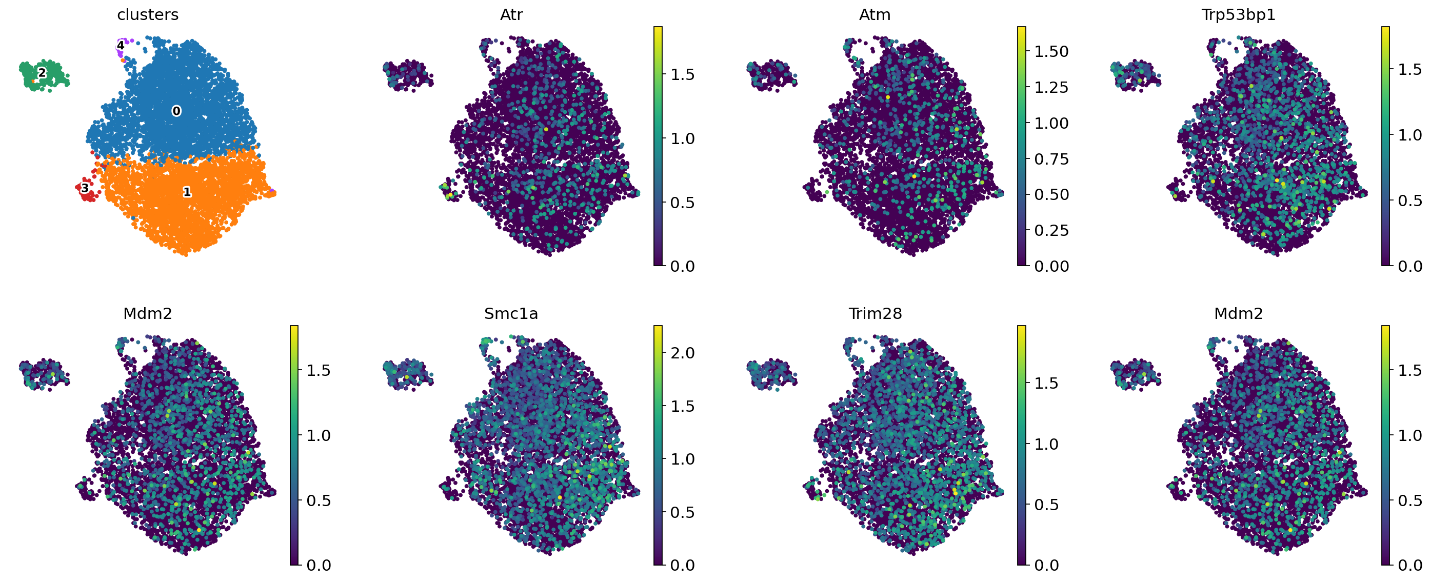
**

**Supplementary Figure 4. Low expression of SASP gene *Mmp2* and DNA damage response genes in α cells of NOD mice. (A)** Dot-plot of *Mmp2* expression across subpopulations and violin plot of *Mmp2* across α cells of mice at different ages. (B) Dot-plots of DNA damage response and repair genes revealed generally low expression in α cells from all clusters.

**Insulitis Residual Islet**


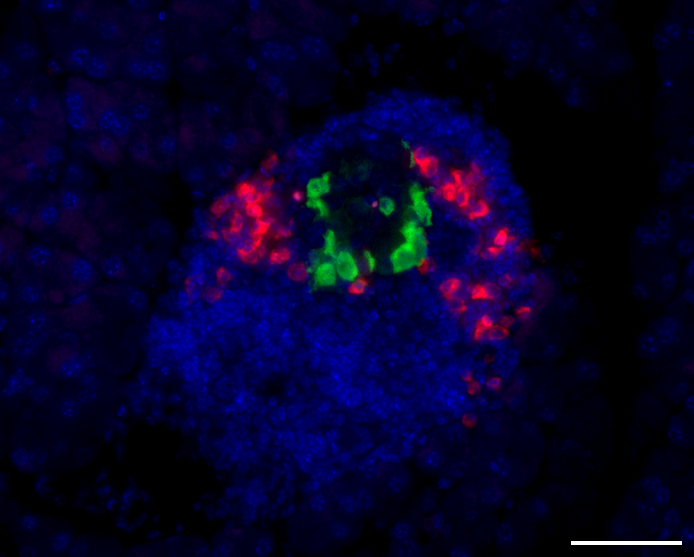
 **
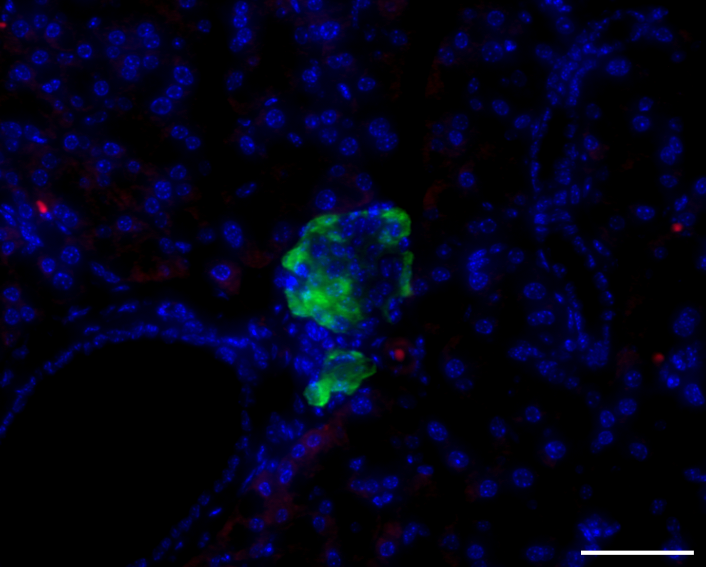
**

CD74

Gcg

DAPI

**Supplementary Figure 5. Immunohistochemistry analysis of CD74 and Gcg in NOD pancreas sections.** CD74 was co-stained with Gcg on pancreas sections from 13-15 week (n=3) euglycemic female NOD mice. Representative images of an islet with insulitis and a residual islet are shown. Scale bars = 50 µm.

**
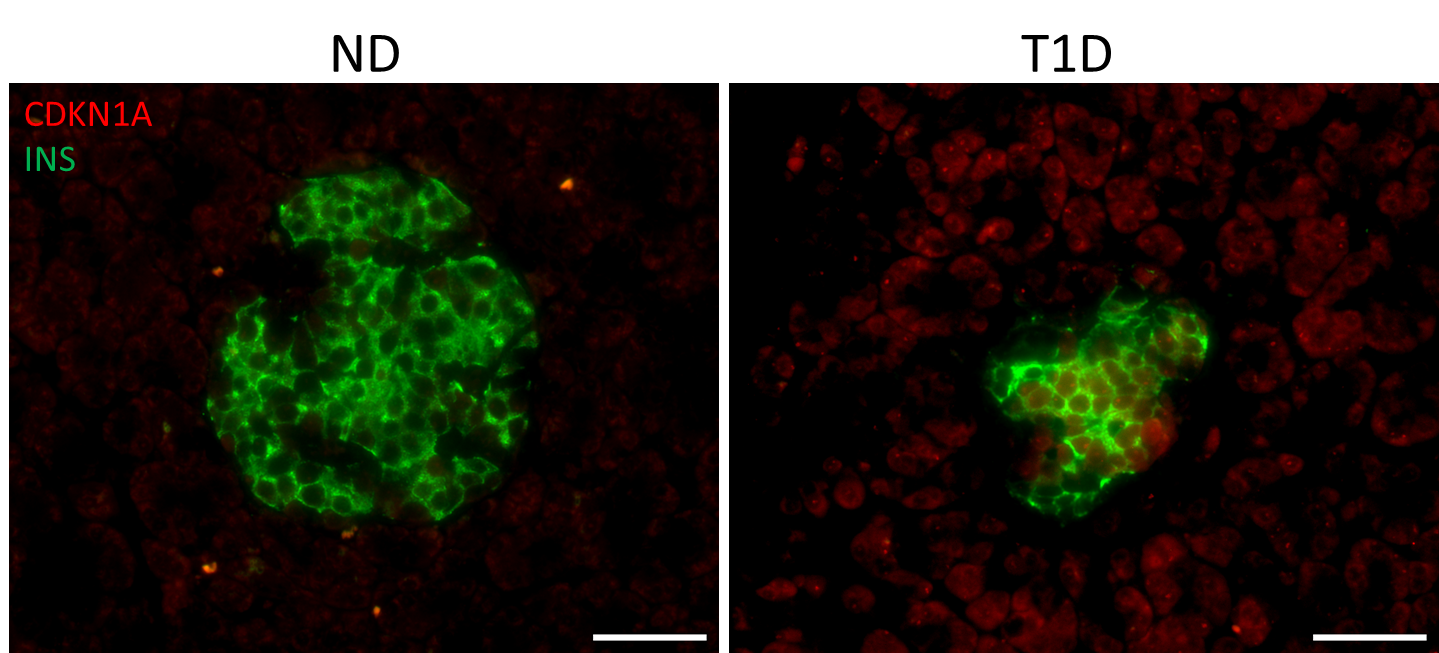
**

**Supplementary Figure 6. Expression of CDKN1A in INS+ cells in T1D.** Representative immunohistochemistry for CDKN1A and INS in nondiabetic (ND, nPOD 6336) and T1D (nPOD 6342) pancreas sections. Donor information is listed in Supplementary Table 1. Scale bars = 50 μm.
